# CavKA^HYBRID^ – between hard spheres and Gaussians

**DOI:** 10.1186/1758-2946-4-S1-P20

**Published:** 2012-05-01

**Authors:** Florian Koelling, Knut Baumann

**Affiliations:** 1 Institut für Medizinische und Pharmazeutische Chemie, Technische Universität Braunschweig, Beethovenstr. 55, D-38106 Braunschweig, Germany

## 

Three dimensional pharmacophores and pharmacophore searches are well established in virtual screening and have been applied successfully in many prospective and retrospective drug discovery campaigns [1]. While the pharmacophore concept offers an easy and abstract understanding of molecular properties, plenty of user intervention is required to define feasible models.

Recently, Silicos NV provided their freely available ligand centric pharmacophore method Pharao [2]. Pharao employs three dimensional Gaussians to reflect a molecule’s pharmacophoric properties, in contrast to most methods which use conventional hard sphere models. Gaussian models show the advantage that they require far less user intervention for model creation.

Here we present a hybrid model that utilizes the Gaussian pharmacophore representation of Pharao to be adapted and used in CavKA (Cavity Knowledge Acceleration), our own in-house strategy for structure based pharmacophore generation. CavKA interprets ligand-receptor complexes and detects interaction between ligand and binding site in order derive pharmacophore models automatically. In addition Grid [3] Molecular Interaction Fields (MIFs) can be used to weight and prioritize interacting features. By combining the smooth nature of Gaussian pharmacophores in the binding site and representing the receptor by a hard sphere excluded volume, structure based pharmacophores can be created without any user intervention.

The performance of CavKA^HYBRID^ is compared to the purely ligand centric approach Pharao in a retrospective virtual screening on the Field Screen [4] dataset. It is the objective of this study to analyse in how far protein information can be used to improve screening results while no user intervention is required for model building. Furthermore the effect of a Gaussian representation of the excluded volume in CavKA^HYBRID^ is analysed.
